# Dr. John L. Van Alstyne

**Published:** 1914-10

**Authors:** 


Dr. John L. Van Alstyne, Albany, 1862, died at his home in Binghamton, May 18, aged about 75.

